# Enantiocomplementary *Yarrowia lipolytica* Oxidoreductases: Alcohol Dehydrogenase 2 and Short Chain Dehydrogenase/Reductase

**DOI:** 10.3390/biom3030449

**Published:** 2013-08-12

**Authors:** Kamila Napora-Wijata, Gernot A. Strohmeier, Manoj N. Sonavane, Manuela Avi, Karen Robins, Margit Winkler

**Affiliations:** 1 ACIB (Austrian Centre of Industrial Biotechnology) GmbH, Petersgasse 14/III, Graz 8010, Austria; E-Mails: kamila.napora@acib.at (K.N.-W.); gernot.strohmeier@acib.at (G.A.S.); manojsonavane4@gmail.com (M.N.S.); 2 Institute of Organic Chemistry, TU Graz, Stremayrgasse 9, Graz 8010, Austria; 3 LONZA AG, Rottenstrasse 6, Visp 3930, Switzerland; E-Mails: m.avi@gmx.net (M.A.); karen.robins@lonza.com (K.R.)

**Keywords:** Zn-dependent alcohol dehydrogenase, *Yarrowia lipolytica*, biooxidation, short chain dehydrogenase/reductase, medium chain secondary alcohols, enantioselective reduction

## Abstract

Enzymes of the non-conventional yeast *Yarrowia lipolytica* seem to be tailor-made for the conversion of lipophilic substrates. Herein, we cloned and overexpressed the Zn-dependent alcohol dehydrogenase ADH2 from *Yarrowia lipolytica* in *Escherichia coli*. The purified enzyme was characterized *in vitro*. The substrate scope for *Yl*ADH2 mediated oxidation and reduction was investigated spectrophotometrically and the enzyme showed a broader substrate range than its homolog from *Saccharomyces cerevisiae*. A preference for secondary compared to primary alcohols in oxidation direction was observed for *Yl*ADH2. 2-Octanone was investigated in reduction mode in detail. Remarkably, *Yl*ADH2 displays perfect (*S*)-selectivity and together with a highly (*R*)-selective short chain dehydrogenase/ reductase from *Yarrowia lipolytica* it is possible to access both enantiomers of 2-octanol in >99% ee with *Yarrowia lipolytica* oxidoreductases.

## 1. Introduction

Chiral alcohols are valuable building blocks for pharmaceuticals and agrochemicals [1] and a multitude of studies have been devoted on biocatalytic methodologies for their production. Nevertheless, there is still a high demand for new enzymes, which operate on specific substrates with high activity and selectivity. Especially, lipophilic compounds are a challenge for classical biocatalysis because substrate availability is low in the aqueous phase in which the enzymes are usually present. The non-conventional yeast *Yarrowia lipolytica* is typically found in lipid-rich media [2] and therefore its enzymes are thought to be evolved to metabolize non-polar substrates [3]. The work of Fantin *et al.* on new alcohol oxidation activities showed, for example, that *Yarrowia lipolytica* alcohol dehydrogenases (ADHs) are highly interesting candidates for biocatalysis [4]. *In vivo*, yeast ADHs are mostly responsible for ethanol formation or consumption and cofactor balance. *In vitro*, ADH1 from *Saccharomyces cerevisiae* (*Sc*ADH1; E.C: 1.1.1.1) is used for cofactor recycling with EtOH as the sacrificial substrate in order to promote NADH dependent enzyme catalyzed reduction [5]. *Sc*ADH1 is a well-studied Zn- and NAD(H) dependent enzyme [6] with known crystal structure (pdb code: 2hcy). The *Yarrowia lipolytica* genome codes for five homologous Zn-dependent ADHs. They are currently filed as putative enzymes [7]. Three of these five proteins were annotated as putative ADH1, ADH2, and ADH3, one as a protein with similarity to putative *Yarrowia lipolytica* ADH3, and one as protein with similarity to mitochondrial ADH3 of *S. cerevisiae*. Of these proteins, ADH2 showed the highest similarity to *Sc*ADH1 and was therefore chosen as a target enzyme (Table 1). *Yl*ADH2 shows sequence similarity to alcohol dehydrogenases from other yeasts [8], e.g., *Pichia stipitis* ADH1 (74% identity) [9], *Candida maltosa* ADH2A [10] (73% identity), *S. cerevisiae* ADH3 (71% identity), and *Hansenula polymorpha* ADH (75% identity) [11]. Whereas *Sc*ADH3-like enzymes from different yeasts are mitochondrial enzymes [3,12], the primary sequences of *Sc*ADH1 and *Yl*ADH2have no mitochondrial targeting sequence according to the PSORTII algorithm [13]. Herein we report the heterologous expression of *Yarrowia lipolytica* ADH2, the enzyme’s substrate scope and its enantioselectivity. Further, *Yl*ADH2 is compared to a *Yarrowia lipolytica* short chain dehydrogenase/ reductase (*Yl*SDR), which is enantiocomplementary and offers the possibility to synthesize the other enantiomer of 2-octanol.

**Table 1:** Protein similarities of *Saccharomyces cerevisiae* ADH1 and *Yarrowia lipolytica* Zn-dependent ADHs. italics: Identities (%), bold: positives (%).

	*Sc*ADH1	*Yl*ADH1	*Yl*ADH2	*Yl*ADH3	*Yl*ADH	*Yl*ADH
Acc. Nr.:	NP_014555	XP_503282	XP_504077	XP_500127	XP_500087	XP_503672
NP_014555	100	*68*	*68*	*69*	*66*	*54*
XP_503282	**80**	100	*94*	*98*	*81*	*57*
XP_504077	**82**	**98**	100	*94*	*79*	*56*
XP_500127	**80**	**99**	**97**	100	*82*	*58*
XP_500087	**80**	**90**	**85**	**90**	100	*53*
XP_503672	**70**	**70**	**70**	**71**	**69**	100

## 2. Results and Discussion

In continuation of our search for versatile oxidoreductases especially for lipophilic compounds [14], we amplified the ADH2 gene from genomic DNA of the *Yarrowia lipolytica* CLIB122 strain and cloned it into two different vector systems. In addition to the native ADH2 sequence, an *N*-terminal His-tag was introduced to facilitate enzyme purification. *Yl*ADH2 expression in the pEHisTEV vector [15] – that adds an *N*-terminal His-tag and a TEV protease cleavage site to the protein of interest – with the T7 promoter resulted in approximately identical expression level compared to untagged *Yl*ADH2 expressed from pMS470, a vector with tac promoter [16] (see Figures S1 and S2 in the supplementary information). His-tagged *Yl*ADH2 was then purified by Ni-affinity chromatography and used for *in vitro* characterization. Investigation of the cofactor specificity revealed, as expected, a strong preference of *Yl*ADH2 for NAD(H) over NADP(H) [6]. We were particularly interested in the substrate tolerance of *Yl*ADH2 and investigated the oxidation of the following substrates: EtOH, 2-propanol, 1-butanol, (2*R*,3*R*)-butanediol, cyclohexanol, 4-methyl-2-pentanol, *rac*-2-heptanol, 1-octanol, *rac*-2-octanol, (*R*)-2-octanol, (*S*)-2-octanol, 1-nonanol, *rac*-2-nonanol, 1-decanol, 1-dodecanol, 1-phenylethanol, (*R*)-2-amino-2-phenylethanol, (*S*)-2-amino-2-phenylethanol, phenylacetaldehyde, adonitol, arabitol, xylitol, sorbitol, and mannitol. Due to the lipophilicity of long chain alcohols, surfactants were used to increase their solubility under assay conditions [17].

**Table 2:** Exploration of the substrate spectrum of *Yl*ADH2.

Entry	Substrate	Relative oxidation activity (%)	Substrate	Relative reduction activity (%)
1	2-propanol	53	acetone	<5
2	1-butanol ^a^	9		
3	*rac*-4-methyl-2-pentanol	6		
4	*rac*-2-heptanol ^a^	64		
5	1-octanol ^b^	7		
6	*rac*-2-octanol ^a^	100 ^c^	2-octanone	100 ^d^
7	1-nonanol ^b^	7		
8	*rac*-2-nonanol ^a^	81	2-nonanone	106
9	1-decanol ^b^	6		
10	*rac*-2-decanol ^a^	77	2-decanone	100

^a^ Tween 20 was used as solubilizer at 0.45% v/v end concentration; ^b^ Tween 20 was used as solubilizer at 0.75% v/v end concentration. 0.75% Tween 20 reduced the activity towards *rac*-2-octanol oxidation by 20% compared to 0.45%; ^c^ 100% corresponds to 1.1 ± 0.1 U·mg^−1^; ^d^ 100% corresponds to 0.50 ± 0.06 U·mg^−1^.

Ethanol is by far the best substrate for ADH1 from *Saccharomyces cerevisiae* [6] and *Yl*ADH2 can also oxidize EtOH, however, its specific activity is two orders of magnitude lower than that of a commercial preparation of *Sc*ADH1 (>300 U/mg as specified by the manufacturer). In this study, the above-mentioned compounds were subjected to both *Yl*ADH2 and *Sc*ADH1 oxidation. Except for EtOH (see above) and 2-propanol (197 ± 77 mU/mg), *Sc*ADH1 showed no significant activity for any substrate. *Yl*ADH2 exhibited a much broader substrate tolerance than its homolog from *Saccharomyces cerevisiae* (Table 2). Substrates which showed less than 5% of the activity towards 2-octanol oxidation are not listed in Table 2. The highest specific activity was observed for the oxidation of racemic 2-octanol (1.1 ± 0.1 U·mg^−1^), which is a value similar to that observed for *Yarrowia lipolytica* short chain dehydrogenase/reductase *Yl*SDR (NCBI Accession Nr. XP_500963.1) [14]. Both enzymes clearly preferred secondary to primary alcohols. However, in contrast to *Yl*SDR, *Yl*ADH2 was not able to oxidize carbohydrate substrates.

**Figure 1:**
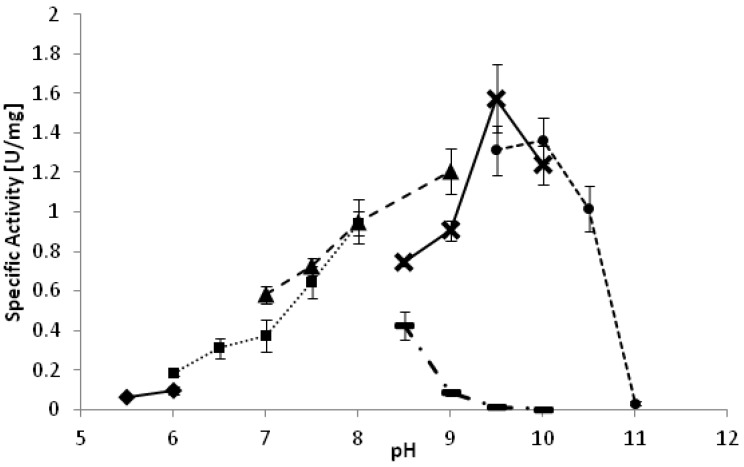
pH optimum of *Yl*ADH2 catalyzed oxidation of (*S*)-2-octanol. ♦: citrate; ■: potassium phosphate; ▲: Tris-HCl; ▬: borate; X: glycine; ●: carbonate.

The optimal reaction temperature for the oxidation of racemic-2-octanol was determined between 25 °C and 37 °C. *Yl*ADH2 showed a plateau of highest activity between 28 °C and 33 °C. The optimal pH of the reaction is strongly dependent on the reaction buffer as depicted in Figure 1. Whereas high activities were observed at pH 9.5 in carbonate and glycine buffer, the same pH was detrimental in borate buffer. A similarly negative effect of borate buffer was also observed for the *Yl*SDR enzyme.

**Figure 2:**
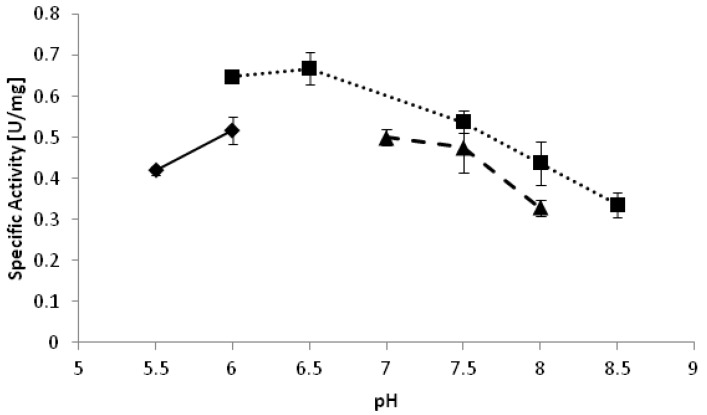
pH optimum of *Yl*ADH2 catalyzed reduction of 2-octanone. ♦: citrate; ■: potassium phosphate; ▲: Tris-HCl.

*Yl*SDR was catalyzing the reduction of several substrates and displayed its highest activity for ribulose [14]. *Yl*ADH2, by contrast, was highly specific for medium chain lipophilic ketone substrates among those tested (see experimental section). Relative specific activities for substrate reduction are shown in Table 2 and the absolute values were approximately 0.5 U·mg^−1^. The optimal pH of the reduction of 2-octanone appeared to be pH 6.5 (Figure 2). Interestingly, reduction reactions often proceed better at relatively low pH as compared to oxidations [18,19]. In the mechanism of a reduction reaction, a hydride is transferred from the nicotinamide donor to the substrate simultaneously to the addition of a proton. At a low pH, the amino acid residues of the protein are predominantly protonated, which facilitates the proton transfer. In oxidation direction, a proton needs to be removed from the substrate, typically from a basic amino acid residue in the active site. In this case, the deprotonated state of the protein at elevated pH seems to be beneficial. 

The determination of kinetic parameters for *Yl*ADH2 mediated oxidation and reduction showed that the *k*_cat_ value for reduction of 2-octanone is approximately half of the value of the respective oxidation (Table 3). 

**Table 3:** Apparent kinetic parameters for *Yl*ADH2.

	Oxidation		Reduction
	(*S*)-2-octanol	NAD^+^	2-octanone
*K*_m_ [mM]	1.42 ± 0.03	17.8 ± 1.26	5.38 ± 0.76
*k*_cat_ [s^−1^]	1.05 ± 0.52	3.43 ± 0.05	0.56 ± 0.04
*k*_cat_/*K*_m_ [s^−1^·mM^−1^]	0.74 ± 0.50	0.19 ± 0.07	0.10 ± 0.16

**Figure 3:**
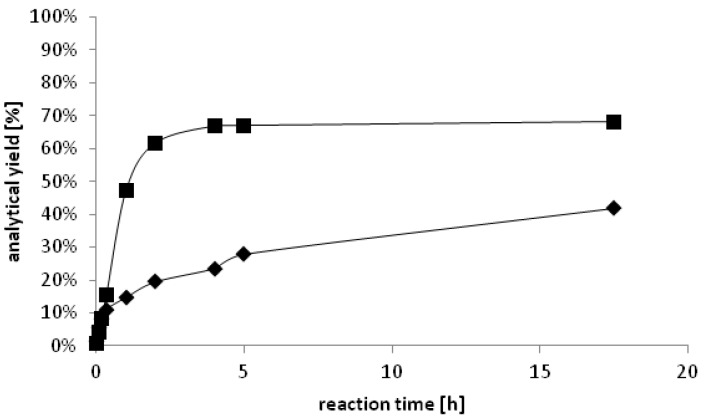
Time dependent formation of ♦ (*S*)-2-octanol (<99% *ee*) catalyzed by *Yl*ADH2 and ■ (*R*)-2-octanol (<99% *ee*) catalyzed by *Yl*SDR.

Alcohol dehydrogenases are often used as catalysts for enantioselective syntheses – on laboratory scale and in industrial processes [20,21]. To determine the enantiopreference of *Yl*ADH2, the single enantiomers of 2-octanol were subjected to NAD^+^ mediated oxidation. The monitored NADH formation was significantly faster in case of (*S*)-2-octanol (1.1 U·mg^−1^) compared to the (*R*)-enantiomer (<0.2 U·mg^−1^) – a first indication for (*S*)-selectivity of the enzyme. In order to verify this result, we investigated the reaction in reduction direction. Therefore, 2-octanone was used as the substrate and the products were analyzed by chiral gas chromatography after derivatization to the corresponding acetates. *Yl*ADH2 produced exclusively the (*S*)-enantiomer (>99% *ee*). By contrast, the (*R*)-enantiomer was obtained in >99% *ee* in case that *Yl*SDR was applied as the biocatalyst (Figure 3) 

**Scheme 1:**
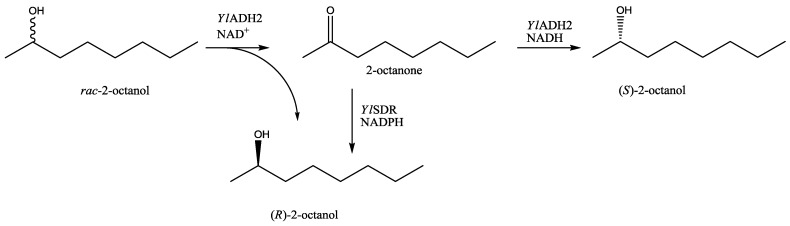
Routes to enantiomerically pure (*S*)- and (*R*)-2-octanol via *Yarrowia lipolytica* oxidoreductases.

In order to improve the conversions, we applied cofactor recycling, using glucose dehydrogenase (GDH) and formate dehydrogenase (FDH) [22] in different combinations of enzyme and co-substrate concentrations. The cofactor recycling system GDH/glucose gave moderate conversions in comparison to FDH/formate. Using 0.02 U of FDH in combination with sodium formate (100 mM) at 0.5 mL scale, 70% of 2-octanone were reduced to (*S*)-2-octanol in >99% *ee* within one hour. After 2.5 h, the conversion was 83% and full conversion (>99% *ee*) was observed after a reaction time of <16 h. Enantiomerically pure lipophilic alcohols can be used as derivatizing agent for the enantioseparation of carboxylic acids [23] or e.g., for the preparation of functional materials. (*S*)-2-Octanol, for instance, was used as the chiral selector in microemulsion electrokinetic chromatography [24]. The (*R*)-enantiomer served as a precursor for chiral liquid crystals [25]. The two *Yarrowia lipolytica* oxidoreductases described herein offer the possibility to produce both enantiomers of 2-octanol in highly pure form, possibly by oxidative kinetic resolution of racemic 2-octanol, or by the reduction of prochiral 2-octanone (Scheme 1).). 

## 3. Experimental

### 3.1. General

*Yarrowia lipolytica* CLIB 122 (supplementary information Figure S3) was obtained from Centre International de Ressources Microbiennes (CIRM, France)*. E. coli* cells were cultivated in RS 306 and Multitron shakers (Infors AG), and the cells were harvested with Avanti centrifuge J-20 (Beckman Coulter). Cell pellets were disrupted with a Branson 102C converter, power was supplied with a Branson Sonifier 250 or a French Press model and cell free extract was obtained by centrifugation in Ultracentrifuge Optima LE80K (Beckman Coulter). Enzymes were purified using a HisTrap^TM^ FF 5 mL column on an ÄKTA Purifier 100 with Frac-950, software Unicorn 4.11, and desalted using a HiPrep^TM^ 26/10 Desalting column on an ÄKTA Prime, software PrimeView 5.0 (GE Healthcare Life Sciences). Protein samples were analyzed with 4–12% NuPAGE^®^ Bis-Tris Gel (Invitrogen) and photometric measurements were carried out on Synergy Mx plate reader (BioTek) using the Gen5.11 Software. Chiral GC analyses were carried out on a Hewlett-Packard 6890 instrument. NADH and NAD^+^ (sodium salt; 97% pure) was obtained from Roche Diagnostics. GDH was obtained from DSM Innovative Synthesis BV. 2-Nonanone and 2-decanone were purchased from Alfa Aesar and all other chemicals including alcohol dehydrogenase from *Saccharomyces cerevisiae* (lyophilized powder, ≥300 U·mg^−1^, order number A7011) were purchased from Sigma–Aldrich/Fluka and used as received. 

### 3.2. Isolation of Genomic DNA and Gene Cloning

Genomic DNA from *Yarrowia lipolytica* strain CLIB 122 was isolated according to the published procedure [26].

The fragment corresponding to *Yl*ADH2 was amplified from genomic DNA using Phusion^®^ High-Fidelity DNA polymerase (Finnzymes) with the following primers: pEHisTEVmutYlADH2_f: 5'-TAC GAG ATA TCA TGT CTG CTC CCG TCA TCC CC-3'; pEHisTEVmutYlADH2_r: 5'-TAA CTG CGG CCG CTT ACT TGG AGG TGT-3'. The *Eco*RV and *Not*I restriction sites are underlined. The gene was cloned into the pEHisTEV vector, previously digested with *Eco*RV and *Not*I. 

*N*-Terminally tagged and untagged *Yl*ADH2 were cloned into vector pMS470 as follows: the fragments were amplified from pEHisTEV:ADH2 using Phusion^®^ High-Fidelity DNA polymerase (Finnzymes) and following primers: pMS470d8ADH2_f 5'-TAT CAC ATA TGT CTG CTC CCG TCA TC-3'; pMS470d8ADH2_r 5'-TTT CTG CAT GCT TAC TTG GAG GTG TC-3'; pMS470d8_HIS-TEVADH2_f 5'-ATA CAT ATG TCG TAC TAC CAT CAC CAT CAC C-3' and pMS470d8_HIS-TEVADH2_r 5'-ATA GCA TGC TTA CTT GGA GGT GTC CAG-3'; The restriction sites *Nde*I and *Sph*I are underlined. Amplification conditions were: 98 °C for 5 min, followed by 30 cycles of 98 °C for 30 s, 55 °C for 30 s, and 72 °C for 30 s, then a final incubation of 72 °C for 7 min. The PCR products were gel separated and the excised DNA was purified with the QIAquick Gel Extraction Kit. The DNA was digested with *Nde*I and *Sph*I restriction enzymes (Fermentas) in the presence of Tango buffer (Fermentas) and column purified according to QIAquick PCR purification protocol. The genes were cloned into the pMS470 vector, previously digested with *Nde*I and *Sph*I and dephosphorylated with Calf Intestine Alkaline Phosphatase (Fermentas) in the presence of FastDigest buffer (Fermentas), using T4 polymerase (Fermentas) in T4 DNA ligase buffer (Fermentas), at room temperature for 1 h.

The fragment corresponding to *Yl*SDR was amplified using Phusion^®^ High-Fidelity DNA polymerase (Finnzymes) with the following primers: YaliSDR2470_f: 5’-AAT CAC ATA TGC CTG CAC CAG CAA CCT AC-3’ and YaliSDR2470_r: 5’-AAT CAG CAT GCT CAA GGA CAA CAG TAG CC-3’. The *Nde*I and *Sph*I restriction sites are underlined. Amplification conditions were: 98 °C for 30 s, followed by 30 cycles of 98 °C for 10 s, 58 °C for 20 s, and 72 °C for 30 s, then a final incubation of 72 °C for 7 min. The PCR products were gel separated and the excised DNA was purified with the QIAquick Gel Extraction Kit (QIAGEN). The DNA was digested with *Nde*I and *Sph*I restriction enzymes (Fermentas) in the presence of Tango buffer (Fermentas) and column purified according to the QIAquick PCR purification protocol. The gene was cloned into the pK470 vector, which contained an *N*-terminal His-Tag (for the vector map, see Figure S4 supplementary information). The pK470 vector was digested with *Nde*I and *Sph*I and gel purified according the procedure described above, prior to the ligation. Ligation was carried out with T4 polymerase (Fermentas) in T4 DNA ligase buffer (Fermentas), at room temperature for 1 h. 

The constructs were transformed into electrocompetent E*. coli* TOP10 F’ cells (Invitrogen) and cells were plated out on LB with 50 μg/mL kanamycin (for pEHisTEV and pK470) or 100 μg/mL ampicillin (for pMS470). The plasmids were isolated with the GeneJET™ Plasmid Miniprep Kit (Fermentas) and the sequences confirmed by LGC genomics. The plasmids were then transformed into electrocompetent *E. coli* BL21 (DE3) Gold cells (Stratagen).

### 3.3. Expression and Purification

Expression and purification of *Yl*SDR was carried out as described previously (supplementary information Figure S5) [14]. *E. coli* BL21 (DE3) Gold harboring ADH2 plasmids were cultivated as follows: overnight cultures [50 mL LB with 50 μg/mL kanamycin (for pEHisTEV) or 100 μg/mL ampicillin (for pMS470)] were inoculated with a single colony and grown overnight at 37 °C in an orbital shaker at 110 rpm. 500 mL LB medium with the appropriate antibiotic in 2-L baffled Erlenmeyer flasks were inoculated to an OD of 0.1. These main cultures were grown at 37 °C and 110 rpm to an OD of 0.4–0.6, cooled on ice for 30 min, induced with 0.5 mM of IPTG and supplemented with 0.25 mM ZnSO_4_ [27]. The cultures were incubated for 20 h at 16 °C and 23 × *g*. The cells were harvested by centrifugation (2,831 × *g*, 4 °C, 10 min), washed with buffer, and disrupted by sonication or French press treatment in Tris/HCl buffer (40 mM; 0.3 M NaCl, pH 8.5). After centrifugation at 72,647 × *g*, 4 °C for 1 h, the cell free extract was either stored at −20 °C or subjected to Ni-affinity chromatography, re-buffered into potassium phosphate buffer (50 mM, 500 mM NaCl, 40 mM KCl pH 8.5), concentrated with Vivaspin 20 (Sartorius Stedim Biotech S.A), shock frozen in liquid nitrogen, and stored at −80 °C. Protein concentrations were determined using the Bradford method. 

### 3.4. Substrate Scope

Alcohol dehydrogenase activity of recombinant *Yl*ADH2 and commercial *Sc*ADH1 were determined by following the reduction of NAD(P)^+^ at 340 nm in UV-Star Polystyrene plates (Greiner Bio-One). Specifically, 20 µL substrate solution (various alcohols and sugars, 100 mM in 50 mM potassium phosphate, 40 mM KCl, pH 8.5) was added to 140 µL potassium phosphate (50 mM, 40 mM KCl, pH 8.5), followed by 20 µL enzyme solution (0.05–0.1 mg/mL; *Sc*ADH1 dissolved freshly in 10 mM sodium phosphate, pH 7.5; purified *Yl*ADH2 was thawed on ice and diluted appropriately). The reaction was started by addition of 20 µL NAD^+^ (or NADP^+^; 10 mM in water) and monitored at 28 °C for *Yl*ADH2 and 30 °C for *Sc*ADH1 for 10 min. The following substrates were investigated: EtOH, 2-propanol, 1-butanol, (2*R*,3*R*)-butanediol, cyclohexanol, 4-methyl-2-pentanol, *rac*-2-heptanol, 1-octanol, *rac*-2-octanol, (*R*)-2-octanol, (*S*)-2-octanol, 1-nonanol, *rac*-2-nonanol, 1-decanol, 1-dodecanol, 1-phenylethanol, (*R*)-2-amino-2-phenylethanol, (*S*)-2-amino-2-phenylethanol, phenylacetaldehyde, adonitol, arabitol, xylitol, sorbitol, and mannitol. To substrates with limited water solubility, 4.5% or 7.5% v/v of Tween 20 was added to the 100 mM substrate stock. In case of phenylacetaldehyde, the addition of 50% DMSO was necessary to ensure a homogenous reaction mixture. Each reaction was performed at least in two sets of quadruple measurements. Blanks without substrate were subtracted. Activity units are defined as the amount of enzyme producing 1 µmol of NADH per min. Specific activity was expressed as units per mg of protein.

The reduction of acetone, cyclohexanone, octanal, 2-octanone, 2-nonanone, 2-decanone, 2-dodecanone, acetophenone, phenylacetaldehyde, ribose, arabinose, xylose, glucose, mannose, lactose, and fructose was monitored at 340 nm *via* the oxidation of NADH in UV-Star Polystyrene plates (Greiner Bio-One). The conditions above were used with the following modifications: 4.5% v/v of Tween 20 was added to the 100 mM substrate stock solution of 2-ketones and 10% Triton to octanal. The reaction was carried out at pH 7.0 and 28 °C and it was started by addition of 20 µL NADH (7.5 mM in water). Activity units are defined as the amount of enzyme consuming 1 µmol of NADH per min.

### 3.5. Determination of pH Optima

Optimal oxidation pH was determined by following the reduction of NAD^+^ as described in section “substrate scope”. (*S*)-2-Octanol was used as the substrate. For the different pH points, the following buffers were used, each in 50 mM concentration containing 40 mM KCl: citrate (pH 5.5–6.0), potassium phosphate buffer (pH 6.0–8.0), TrisHCl (pH 7.0–9.0), borate (pH 8.5–10.0), glycine (pH 8.5–10.0), and carbonate buffer (pH 9.5–11.0). Similarly, the optimal pH for reduction was determined using 2-octanone as the substrate with above mentioned buffers.

### 3.6. Determination of Kinetic Parameters

The kinetic parameters for (*S*)-2-octanol oxidation and 2-octanone reduction as well as NAD^+^ reduction were determined. (*S*)-2-octanol was used in concentrations from 0.5 mM to 15 mM and assayed in potassium phosphate buffer (50 mM, 40 mM KCl, pH 8.5). 2-Octanone was used in concentrations from 1 mM to 40 mM and assayed in potassium phosphate buffer (50 mM, 40 mM KCl, pH 7.0). The stock solutions contained 4.5% Tween 20 or less. Kinetic parameters for NAD^+^ were determined by oxidation of (*S*)-2-octanol (10 mM) in potassium phosphate buffer (50 mM, 40 mM KCl, pH 8.5) with concentrations of NAD^+^ from 200 µM to 50 mM. All assays were performed as described in section “substrate scope” at 28 °C. The results were evaluated based on Michaelis - Menten kinetics, using SigmaPlot™ version 11.0 for non-linear fitting. 

### 3.7. Determination of Enantioselectivity

**(*S*)-2-octanol** was prepared under the following conditions: purified *Yl*ADH2 in potassium phosphate buffer (50 mM, containing 40 mM KCl, pH 6.5) was mixed with 2-octanone (100 mM in the same buffer with 7.5% v/v Tween 20) and NADH (100 mM in water) to give 0.16 mg/mL, 10 mM and 11 mM end concentration, respectively, in total volumes of 500 µL. The reaction proceeded at 28 °C in an Eppendorf Thermomixer at 600 rpm. For each time-point, an extra sample was sacrificed. Substrate and products were extracted into 500 µL of ethyl acetate. A triethylamine-4-(dimethylamino)-pyridine stock solution [TEA-DMAP stock solution: DMAP (8.9 mg, 73.2 µmol) dissolved in TEA (2.00 mL, 14.3 mmL)] was added to the sodium sulfate-dried ethyl acetate extract of the reaction (500 µL, contains ≤ 10.0 mM of 2-octanol) (TEA: 68.3 µL, 490 µmol, 98 equivalents.; DMAP: 0.31 mg, 2.5 µmol, 0.5 equivalents) and acetic anhydride (23.6 µL, 250 µmol, 50 equivalents). After keeping the mixture at 40 °C for 3 h, the reaction was quenched by adding saturated sodium chloride solution (300 µL) and subsequent vigorous shaking. Finally, the ethyl acetate layer was directly subjected to GC analysis on a Chirasil-Dex CB column (25 m × 0.32 mm; 0.25 µm film; Varian). The GC settings were as follows: injector 220 °C; 1.0 bar constant pressure H_2_ flow; temperature program: initial temperature 60 °C, 85 °C/rate 1.5 °C per min, hold 3 min; The absolute configuration of 2-octanol was assigned by comparison of the elution order on chiral GC with literature known data [28] and by derivatization of commercial (*R*)- and (*S*)-2-octanol. Retention times were 8.0 min for 2-octanone, 13.9 min for (*S*)-octan-2-yl acetate and 17.2 min for (*R*)-octan-2-yl acetate. 

For cofactor recycling, the same conditions as describe above were used, with the exception that also 0.03 U of FDH and 100 mM of sodium formate were added to the reaction and the content of NADH cofactor was reduced to 1 mM.

**(*R*)-2-octanol** was prepared under the following conditions: purified *Yl*SDR in citrate buffer (50 mM, pH 5.0) was mixed with 2-octanone (100 mM in the same buffer with 3% v/v Tween 20) and NADPH (100 mM in water) to give 0.11 mg/mL, 10 mM and 11 mM end concentration, respectively, in total volumes of 500 µL. The reaction proceeded at 28 °C in an Eppendorf Thermomixer at 600 rpm. Workup and analysis were carried out as described for (*S*)-2-octanol.

## 4. Conclusions

In conclusion, we have shown that *Yarrowia lipolytica* harbors versatile oxidoreductases that catalyze selective oxidation and reduction reactions. From the two enzymes described herein, secondary alcohols are the preferred substrates in the oxidation direction compared to primary alcohols and aldehydes. Medium chain length ketones with the carbonyl function at position C-2 are reduced to the corresponding secondary alcohols in enantio-complementary form: whereas *Yl*ADH2 produced the (*S*)-enantiomer in >99% *ee*, the (*R*)-enantiomer was obtained with *Yl*SDR.

## Supplementary Files

Supplementary File 1 Supporting Information (PDF, 627 KB)
