# Supplementary material for: Enantiocomplementary Yarrowia lipolytica Oxidoreductases: Alcohol Dehydrogenase 2 and Short Chain Dehydrogenase/Reductase
Source: Biomolecules. 2013 Aug 12;3(3):449–60. doi: 10.3390/biom3030449 (PMC4030946; doi:10.3390/biom3030449)
Supplement: Supplementary File 1 — Supporting Information (PDF, 627 KB) [file biomolecules-03-00449-s001.pdf]

Supporting Information

Enantiocomplementary *Yarrowia lipolytica* Oxidoreductases: Alcohol Dehydrogenase 2 and Short Chain Dehydrogenase/Reductase

Kamila Napora-Wijata, Gernot A. Strohmeier, Manoj N. Sonavane, Manuela Avi, Karen Robins and Margit Winkler

Figure S1. *Y. lipolytica* CLIB 122 on YPD medium.

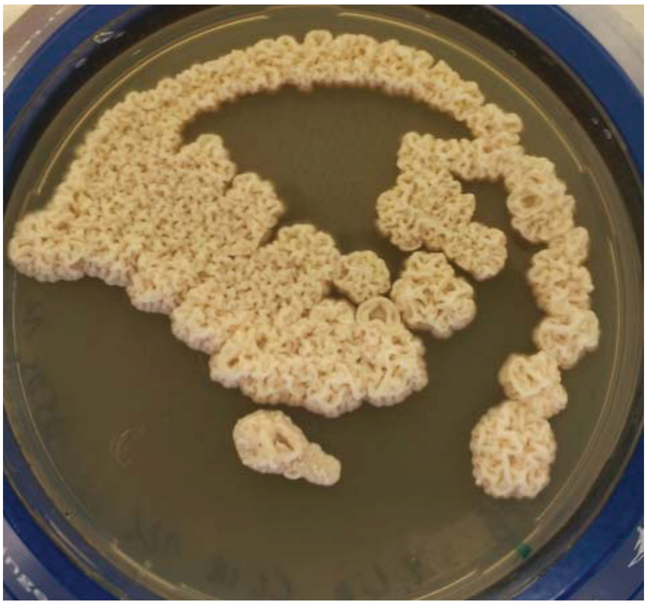

Figure S2. NuPAGE gel of *Yl*ADH2 and *Yl*HisTevADH2 cell free extracts and purified *Yl*HisTevADH2. Lane 1: PageRuler Prestained Protein Ladder (Fermentas); Lane 2: pMS470:d8 vector control with stuffer fragment; Lane 3: pMS470:*Yl*ADH2; Lane 4: pMS470:*Yl*HTADH2; Lane 5: pEHisTEV:*Yl*ADH2; Lane 6: pEHisTEV vector control; Lane 7: purified *Yl*ADH2; expected size of *Yl*ADH2 is 37.3 kDa and tagged *Yl*ADH2 40.8 kDa.

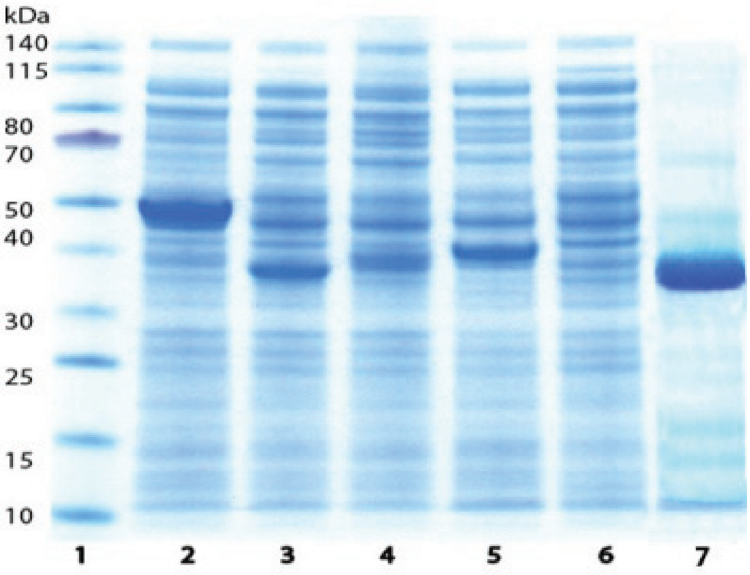

Figure S3. NuPAGE gel of *Yl*HisSDR. Lane 1: PageRuler Prestained Protein Ladder (Fermentas); Lane 2: pK470:*Yl*HisSDRcell free extract; Lane 3: pK470:*Yl*HisSDR cell debris; Lane 4: pK470:*Yl*HisSDR flow through from purification; Lane 4: purified *Yl*HisSDR; expected size of tagged *Yl*SDR is 31.9 kDa.

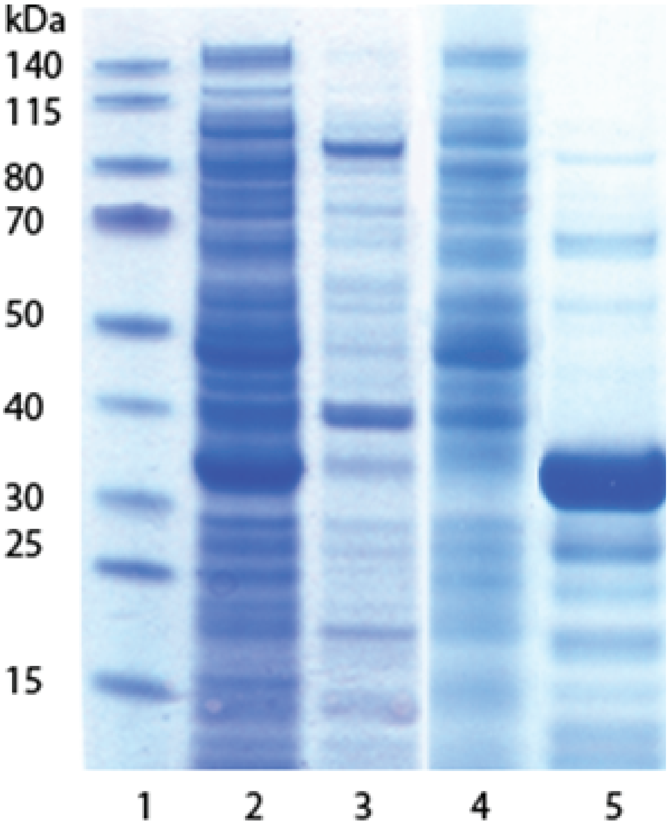

Figure S4. pMS470:*Yl*HisTevADH2 vector map.

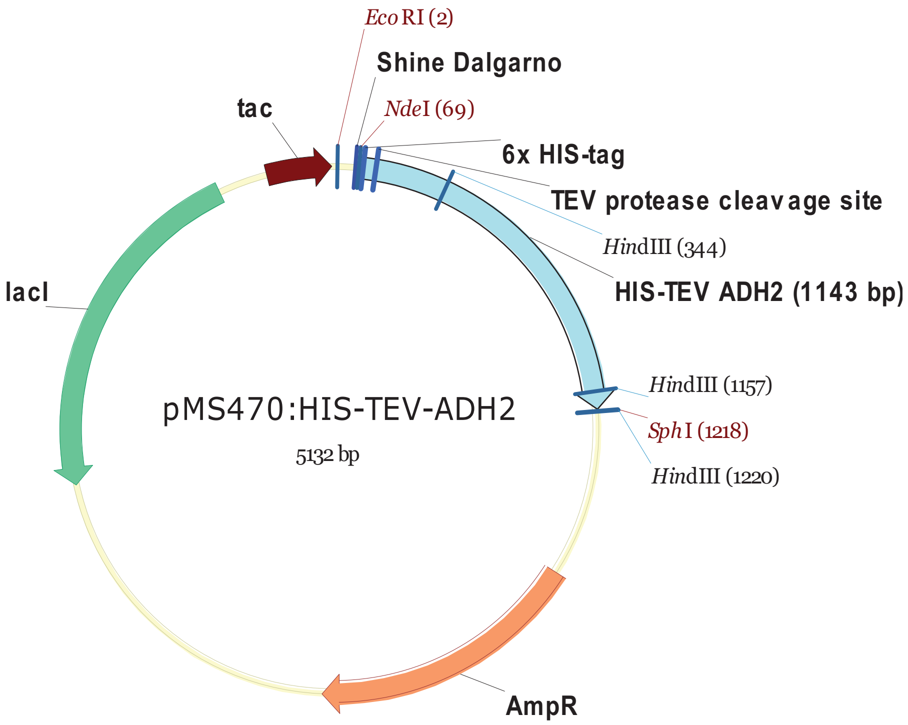

Figure S5. pK470:*Yl*HisSDR vector map.

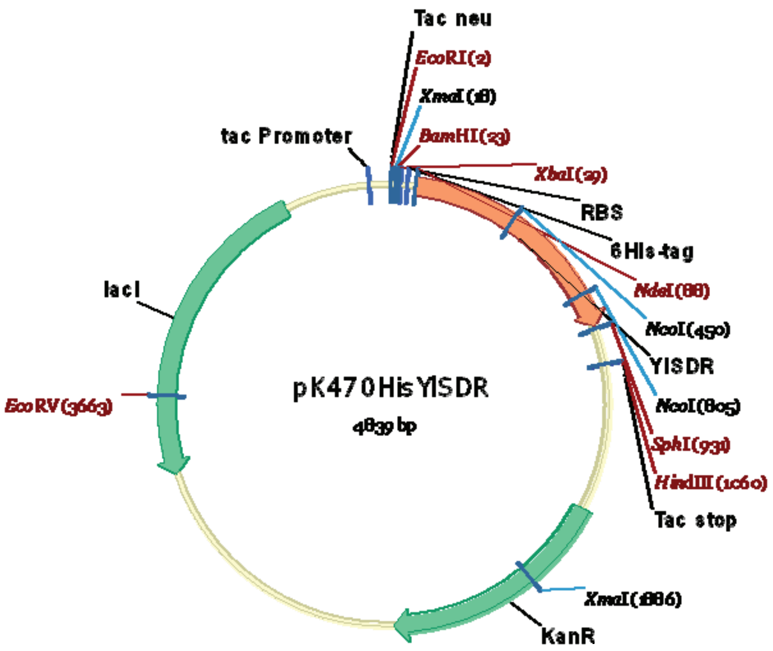

© 2013 by the authors; licensee MDPI, Basel, Switzerland. This article is an open access article distributed under the terms and conditions of the Creative Commons Attribution license (http://creativecommons.org/licenses/by/3.0/).
